# Targeting Histone 3 Variants Epigenetic Landscape and Inhibitory Immune Checkpoints: An Option for Paediatric Brain Tumours Therapy

**DOI:** 10.2174/1570159X21666230809110444

**Published:** 2024-08-15

**Authors:** Sarasa Meenakshi, Krushna Ch Maharana, Lokesh Nama, Udaya Kumar Vadla, Sameer Dhingra, Velayutham Ravichandiran, Krishna Murti, Nitesh Kumar

**Affiliations:** 1 Department of Pharmacy Practice, National Institute of Pharmaceutical Education and Research, Hajipur, Vaishali- 844102, Bihar, India;; 2 Department of Pharmacology & Toxicology, National Institute of Pharmaceutical Education and Research, Hajipur, Vaishali- 844102, Bihar, India

**Keywords:** Epigenetics, histones, pediatric brain tumor, immune checkpoints, immunotherapy, cell signalling

## Abstract

Despite little progress in survival rates with regular therapies, which do not provide complete care for curing pediatric brain tumors (PBTs), there is an urgent need for novel strategies to overcome the toxic effects of conventional therapies to treat PBTs. The co-inhibitory immune checkpoint molecules, *e.g*., CTLA-4, PD-1/PD-L1, *etc*., and epigenetic alterations in histone variants, *e.g*., H3K27me3 that help in immune evasion at tumor microenvironment have not gained much attention in PBTs treatment. However, key epigenetic mechanistic alterations, such as acetylation, methylation, phosphorylation, sumoylation, poly (ADP)-ribosylation, and ubiquitination in histone protein, are greatly acknowledged. The crucial checkpoints in pediatric brain tumors are cytotoxic T lymphocyte antigen-4 (CTLA-4), programmed cell death protein-1 (PD-1) and programmed death-ligand 1 (PD-L1), OX-2 membrane glycoprotein (CD200), and indoleamine 2,3-dioxygenase (IDO). This review covers the state of knowledge on the role of multiple co-inhibitory immunological checkpoint proteins and histone epigenetic alterations in different cancers. We further discuss the processes behind these checkpoints, cell signalling, the current scenario of clinical and preclinical research and potential futuristic opportunities for immunotherapies in the treatment of pediatric brain tumors. Conclusively, this article further discusses the possibilities of these interventions to be used for better therapy options.

## 1 INTRODUCTION

Despite the rarity, the incidence of brain tumors (BT) is found to be higher in children compared to adults. However, there are also tumors other than solid tumors in children. Moreover, it is the second most form of cancer, which represents about 23% of total cancer incidents and is also a leading cause of death among all pediatric cancer patients in the United States. It is reported that the rate of occurrence of brain and nervous system cancer is about 1/161 individuals, and 23,770 new cases of both genders have been diagnosed recently, with 16,050 deaths. Despite the advancement in brain cancer therapy, the death rate has remained the same for the past 30 years, and the accurate cause of BTs development is not yet fully understood. PBTs are entirely different from adult BTs in their location and behaviour as children differ due to alterations in physique and anatomy of the brain, which are still in developing mode. In addition, the treatment options are also to be varied with respect to their age because children show relatively better prognoses than adults under similar conditions.

Based on cell structure, composition, growth rate, and other biological characteristics, BTs are classified into astrocytoma, medulloblastoma (MB), ependymoma, atypical teratoid rhabdoid tumor (ATRT), brain stem glioma, choroid plexus carcinoma, choroid plexus papilloma, craniopharyngioma, diffuse intrinsic pontine glioma (DIPG), desmoplastic infantile astrocytoma, cysts, germ cell tumors, neurofibromatosis, oligodendroglioma, optic glioma, primitive neuroectodermal tumor (PNET), *etc*. However, their names and classification might be altered over time with respect to the availability of data regarding alterations in the tumors.

Local tumors escape from antigen-stimulated primary immune response mediated by major histocompatibility complex (MHC) and T-cell receptor (TCR), followed by co-stimulatory secondary T-cell response mediated by CD28 and CD80/CD86 that can be regulated by inhibitory immune checkpoints by inducing FoxP3+Tregs as reported in different adult malignancies [1, 2]. Apart from FoxP3+ Tregs, myeloid-derived suppressor cells (MDSCs) [3] and tumor-infiltrating Tregs [4] contribute to the cessation of classic and anti-tumor immunities and facilitates the secretion of soluble membrane-bound immunosuppressive molecules [3]. Epigenetics plays an important role in affecting the progression of tumor pathogenesis. Generally, the immune system differentiates the abnormal cells inside the body; eventually, the immune cells identify and eliminate the foreign entity. This process of selection is done by the immune checkpoints [5]. These immune checkpoints act as the control entity that protects the normal cells from damage caused by the immune cells. During tumors, these checkpoints get disturbed and foster the normal immune reaction. The epigenetic modulation of the immune checkpoints can be regulated by various mechanisms like covalent modification, histone modification, and microRNA [6]. The epigenetic functional classification system classifies cancer genes into epigenetic mediators, *i.e*., progenitor cells, modifiers, and modulators, which affect the tumor environment inside the body. Immune modifiers can act on the genes and regulate them on and off [7]. The modulation of the immune checkpoints by epigenetics thus can be a major therapeutic target. In the development of PBT, the epigenetics of histone H3 plays a vital role. Similar to histone H3, numerous important amino acids, such as serine and lysine-14, undergo alterations, including ubiquitination and phosphorylation, thus affecting the progression of the tumor [8]. However, this concept is poorly understood regarding how they contribute to tumor formation. Together, the initiation, metabolism, angiogenesis, and metastasis of cancer genes are influenced by histone 3 lysine modification [9]. The characteristics and results of PBT carcinogenesis can be significantly altered by altering the histone 3 lysine. This novel concept has been used in clinical trials and may possibly result in effective treatments for tumor patients [10].

Moreover, these checkpoints can actively participate in peripheral immune tolerance and also demonstrate a promising role in immune evasion in tumor microenvironments. However, limited research is focused on the potential benefits of these inhibitors against solid tumors in children.

Furthermore, epigenetic regulations in protein-coding genes at both DNA and expression levels determine cell fate as well as organism development. Though epigenetic changes inherit somatically, they are quite reversible and also ensure the timely expression of genes that are naturally involved in the development and differentiation of cells. Also, they promptly express a version of unnatural genes that are not encoded by DNA in response to environmental stimuli for adaptation. They bring notable molecular and structural changes in DNA without altering the sequence [11]. Among the different epigenetic regulations, DNA methylation is a well-studied process that mainly occurs in cytosine residues of CpG islands by DNA methyltransferases (DNMTs). DNA methylation in DNA repeats aids chromosomal stability and inhibits homologous recombination [12], whereas its episode in the genes prevents turbulent transcriptional initiation [13]. Furthermore, global DNA hypomethylation in gene bodies and hypermethylation of CpG islands in the tumor suppressor genes (TSGs) located in the promoter regions are ultimately responsible for the development of cancer [14]. In normal tissues, which are actively involved in the development and differentiation, the CpG islands of most of the gene promoters remain unaltered [15] except for the inactivation of the X-chromosome [16]. However, hypermethylation of CpG islands initially recruits methyl-CpG binding domain (MBD) proteins, which further promotes the recruitment of histone-modifying and chromatin-remodelling complexes to the methylated promoter region, which in turn, silences the target gene [17, 18].

In addition, histone proteins are the hotspots and are highly susceptible to a wide range of epigenetic changes, which, in turn, regulate the chromatin structure and dynamics. These histone proteins (H2A, H2B, H3, and H4) are highly conserved from primitive eukaryote yeast to evolutionarily high-ranked organisms, such as humans. In the nucleus, histone octamer (two of each histone protein) is wrapped by DNA and forms a nucleosome core, which allows the packing of large DNA into the compact chromatin. In this process, the nucleosomal histone core exposes its amino-terminal tail called “histone code”, which is highly prone to endure an array of posttranslational modifications (PTMs) and subsequently reports the genes to express or silence. Aberrant mutations in such vital proteins drive the development of cancer.

Recently, a novel concept has been proposed that the aberrant epigenetic alterations inducing chromatin structural deformities may exhibit acquired chemo-resistance to some extent in cancer patients [19]. To oppose this, the Food and Drug Administration (FDA) has approved different epigenetic therapies for cancers, such as leukemia, which include DNA methylating agents and histone deacetylase inhibitors (HDIs). Moreover, their combinations were also tested in clinical trials [20]. However, in the treatment of glioblastoma (GBM), only HDIs entered into clinical trials but not demethylating agents. Probably, this is because of methylated O^6^-methylguanine DNA-methyltransferase (*MGMT)* promoter (typically involved in DNA repair) sensitizes the tumors to alkylating agents like temozolomide (TMZ), a standard drug for GBM [21]. In general, *MGMT* neutralizes TMZ, which, in turn, leads to the development of resistance in GBM. However, this mechanism is not yet fully understood in children [22-25]. Later, it was predicted that a large frequency of *MGMT* methylation might be attributed to the H3.3 G34R/V (glycine to arginine or valine substitution at position 34 on histone H3.3 variant) subtype but not to K27M (lysine to methionine substitution at position 27) mutated tumors [26], which may aid in poor clinical response to TMZ in most of the high-grade gliomas (HGGs), including DIPG [27-30]. Moreover, demethylating agents may activate oncogenes besides the TSGs because of their non-specific mechanism of action. Furthermore, they may affect genome stability by inducing demethylation in gene bodies and hypomethylation in repetitive sequences. Despite the reluctance of conventional therapies against pediatric HGGs, epigenetic modifying agents that targets histone demethylases (HDMs) and HDIs remain effective against DIPG [31, 32] by inducing the restoration of H3K27me3 in alternative ways, highlighting their synergistic effect in combination [31]. With this brief literature, here, we have critically reviewed different immune checkpoints and their inhibitors in clinical trials as well as the epigenetic landscape of histone proteins (H3) and possible epigenetic therapies for PBTs collectively.

## 2 IMMUNE CHECKPOINTS AND PRECLINICAL STUDIES

### 2.1 PD-1 and PD-L1

**Fig. (1):**
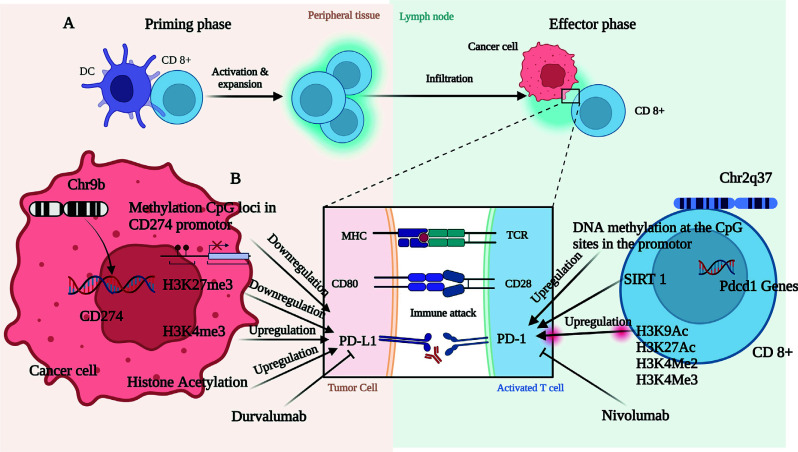
Epigenetic control of PD-1 and the PD-L1 involved in the immunogenic checkpoints in the cancer. Legend: Figure showing an underlying mechanism of epigenetic alteration of PD-1 and PD-L1 involving the immunogenic checkpoints in cancer. (**A**) The priming phase, where the T-cells (CD-8) get activated by the dendritic cells; then, the infiltration of the T-cells occurs. (**B**) An interaction between cancer cells and the T-cells, where cancer cells disturb the regulation of PD-L1. *Durvalumab* blocks the expression of PD-L1; on the other hand, activation of T-cells epigenetically affects the expression of PD-1. *Novolumab* acting as a blocker and normalizing the expression of PD-1 in T-cells.

PD-L1 is a 40 kDa glycoprotein that belongs to type-1 transmembrane glycoproteins and is located on the surface of hematopoietic and parenchymal cells [33]. It is a co-stimulatory molecule belonging to B7-family and is also named B7-H1. It typically interacts with the PD-1 protein, a receptor on activated T and B-cells to regulate both humoral and cell-mediated immunities [1]. PD-1 plays a crucial role in autoimmunity and peripheral immune tolerance. Induction of PD-L1 enables tumor growth by causing immune evasion at the tumor microenvironment [1] and further promotes metastasis and the refractory state of the disease in adult malignancies [34, 35]. PD-L1 expression and its blocking responses vary based on the stage and subtype of the tumor [36, 37]. The low PD-L1 expression in human primary osteosarcoma tumors and high PD-L1 expression in metastatic disease neutralize the response of tumor-infiltrating lymphocytes (TILs) effector by interacting with PD-1+, CD8+, and T-cells [36]. However, targeting PD-L1 with specific antibodies showed better survival benefits in the murine model by restoring lymphocyte anti-tumor functions. Likewise, in a study on a murine model of MB subtypes, the PD-1 blockade was more efficient in group-3 MB with a higher CD8+T to PD-1+T-cells ratio compared to Shh-subtype MB [37], indicating the crucial role of immune divergence in disease outcome or therapy (Fig. **1**).

In pediatric cancers, the differential expressions of PD-L1 between moderate to high [38-42] are due to variations in the available PD-L1 antibody specificity [43]. Moreover, these expressions are positively correlated with survival rates [39]. In preclinical studies on pediatric retinoblastoma and neuroblastoma patient cells, the PD-L1 expression was significantly upregulated in the response to immune stimuli [44, 45]. However, PD-L1/PD-1 specific antibody therapy alone was insufficient to induce an anti-tumor T-cell response in neuroblastoma of murine model, but in combination with BLZ945, a selective inhibitor of colony stimulating factor (CSF)-1 receptor, it was able to block MDSCs induced tumor progression [46], suggesting the reliability of combinational therapies.

FDA approved multiple mAbs against PD-1 and PD-L1, which have survival benefits in a wide spectrum of malignancies [47-51]. Nivolumab, a PD-1 inhibitor, was approved by FDA in spite of a few immune-related adverse events (IRAEs) linked to grade 3-4 toxicities in 16% of melanoma patients. However, with the combination of ipilimumab, which is an inhibitor of cytotoxic T-lymphocyte antigen (CTLA)-4, this toxicity was further raised to 55% [51]. Hence, phase I/II trials are in progress to elucidate the safety dosages of nivolumab alone or in combination with ipilimumab for anti-tumor activity. Due to unresponsiveness to the treatment, the PD-L1 levels are not at all considered for tumor prognosis [47, 52]. Despite the lack of clinical studies on testing the potentiality of blockade of PD-1/PD-L1 in pediatric intracranial solid tumors, a recent report has shown the therapeutic efficacy of nivolumab in recurrent multifocal GBM and biallelic mismatch repair deficiency in children [53]. Phase II clinical trials (NCT02550249) in Spain have been testing an adjuvant role of nivolumab in primary or recurrent GBM patients of >1 year of age. In addition, the European basket trial (NCT02813135) has been testing nivolumab in combination with cyclophosphamide against refractory tumors in patients with ≤ 18 years of age, in the presence or absence of radiation. Similarly, phase I/II clinical trials have been initiated by Children’s Oncology group for testing the safety of nivolumab in combination with ipilimumab against refractory solid tumors in pediatric patients (NCT02304458). In Canada, phase II clinical trials have been planned to test the safety and efficacy of durvalumab (PD-L1 inhibitor) in combination with tremelimumab (CTLA-4 inhibitor) against advanced rare tumors in ≥16 years aged patients (NCT02879162). Likewise, another PD-1 inhibitor, pembrolizumab, was also tested in phase II clinical trials for its safety and dosage limits against recurrent or refractory HGG and DIPGs and was found to be active *via* the PBT consortium. However, there are other clinical trials testing nivolumab alone or in different combinations, such as phase III trials of nivolumab (Opdivo^®^) + ipilimumab (Yervoy^®^) against recurrent GBM (NCT02017717) and nivolumab + radiation *vs.* TMZ (Temodar^®^) in newly-diagnosed GBM (NCT02617589), *etc*. Finally, Ludwig Cancer Research has been sponsored for phase II trials to test the efficacy of durvalumab (MEDI4736) against GBM (NCT02336165) and phase I trials to test the durability of nivolumab with the combination of a dendritic cell (DC), a vaccine against recurrent glioma, astrocytoma, or GBM (NCT02529072).

### 2.2 Cytotoxic T-lymphocyte Antigen-4

It is a homodimeric intracellular glycoprotein belonging to the immunoglobulin (Ig) gene superfamily. However, in response to continuous stimuli and abundant levels of IL-2, CTLA-4 can be exported to the cell surface of different subsets of T-cells, including CD4+, CD8+, and Tregs. CTLA-4 extracellular domain has shared 30% homology with CD28, which is expressed on the surface of T-cells; therefore, it competes with CD28 to interact with B7 family proteins like CD80 (B7-1) or CD86 (B7-2) expressed on antigen-presenting cells (APCs) with high affinity [54, 55]. In this manner, CTLA-4 hampers the production of IL-2, IL-4, IFN-γ, and expression of IL-2R, which results in the depletion of T-cell activation and expansion. Furthermore, it also attenuates the cell cycle to induce death in activated T-cells [56]. CTLA-4 substantially regulates immune tolerance and homeostasis *via* circulatory Tregs, while its deficiency causes systemic lymphoproliferation [57].

However, its direct inhibition with anti-CTLA-4 antibodies dramatically reduced Tregs count and induced expansion of effector CD8+ T-cells at the tumor microenvironment in colon adenocarcinoma [58], further unveiling the therapeutic potential of targeting CTLA-4 [59]. Similarly, when osteosarcoma cells were incubated with recombinant B7-1 or B7-2 ligands, CTLA-4 directed caspase-dependent apoptosis in these cells, further exploring its sensitivity to be targeted by anti-cancer agents. Polymorphism in the CTLA-4 gene (rs231775) increased malignancy in osteosarcoma and Ewing sarcoma since the affinity with B7-1 molecule was significantly increased and concurrently, effector T-cell function was diminished [60, 61]. A large number of preclinical studies have targeted CTLA-4 in adult malignancies, but it remains unexplored in pediatric tumors [62-64]. A report has sown an upregulated expression of CTLA-4 on the surface and cytoplasmic compartments of different pediatric cancer cells of neuroblastoma, osteosarcoma, and rhabdomyosarcoma [65].

However, ipilimumab was the first checkpoint inhibitor studied in phase I trials against 33 different pediatric solid tumor patients (melanoma, sarcoma, renal/bladder carcinoma, and neuroblastoma) aged between 2.4 to 21 years [66], out of which the majority of patients developed immune-related toxicities as described in adult pancreatitis, pneumonitis, colitis, endocrinopathies, and transaminitis, but the overall survival rate was improved by 93.0%. In continuation, ipilimumab and tremelimumab alone or in combination with standard drugs have increased survival benefits by 87.8% in advanced melanoma [49, 51, 67, 68]. However, it demonstrated grade 1-2 IRAEs in the skin and gastrointestinal tract (GST) and grade 3-4 IRAEs in GST, liver, and endocrine system in 1/3 of patients, and death in 1-2% of overall patients. Recently, a phase II study of ipilimumab against stage III/IV melanoma of pediatrics aged between 12-17 years (NCT01696045) was terminated due to underperformance.

### 2.3 CD200/OX2

CD200 is another immune checkpoint abundantly expressed in lymphoid and neuronal tissues [69]. It is a type I transmembrane protein belonging to the B7-family and is involved in T-cell signaling *via* its receptor CD200R, which is expressed on APCs and T-cells. CD200 signaling in macrophages impedes IL-2 and IFN-γ production [70, 71] and reduces effector T-cell response by inducing Tregs [72, 73]. Its expression on the surface of tumor cells directs their immune evasion [73, 74]. Moreover, its overexpression in acute myeloid leukemia was coupled with increased Tregs and reduced natural killer (NK) cells, ultimately resulting in a poor prognosis [75, 76]. Macrophage lineage cells, including brain microglia, displayed an active phenotype and were more abundant in the absence of CD200. Upon facial nerve transection, CD200-deficient neurons induced a rapid microglial response. Furthermore, CD200R agonists reduced mouse and human myeloid cell activity *in vitro*, establishing a dose-response relationship between receptor expression and cellular suppression [70, 71].

The profiling of its expression was studied in different PBTs and it was reported that CD200 protein levels were elevated in ependymoma, MB, and DIPG compared to normal brain tissue [77]. Likewise, its mRNA levels were found to be upregulated in supratentorial ependymoma and group 4 MB subtypes compared to posterior fossa ependymoma and Shh/group 3 MB subtypes, respectively. Preclinical studies on pediatric neuroblastoma patient samples have confirmed that the high expression of CD200 on their cell surface was correlated with reduced effector Th1 response, which was attributed to low IL-2 and IFN-γ levels [78]. Upon anti-CD200 antibody treatment, the Th1 response was restored, thus suggesting the tumor-inducing property of CD200. Similarly, upon CD200 antagonist (OVA+poly-ICLC) treatment, the antigen-specific T-cell mediated immunity and survival rates were substantially enhanced in the murine glioma model [77]. However, there is a gap in currently active clinical trials for both pediatric and adult targeting CD200. An early report in 2011 has shown moderate efficacy of anti-CD200 mAb (samalizumab) against adult B-cell chronic lymphocytic leukemia or multiple myeloma, in spite of few side effects like fatigue, fever, rash, and neutropenia [79].

### 2.4 Indoleamine 2,3-dioxygenase (IDO)

**Fig. (2):**
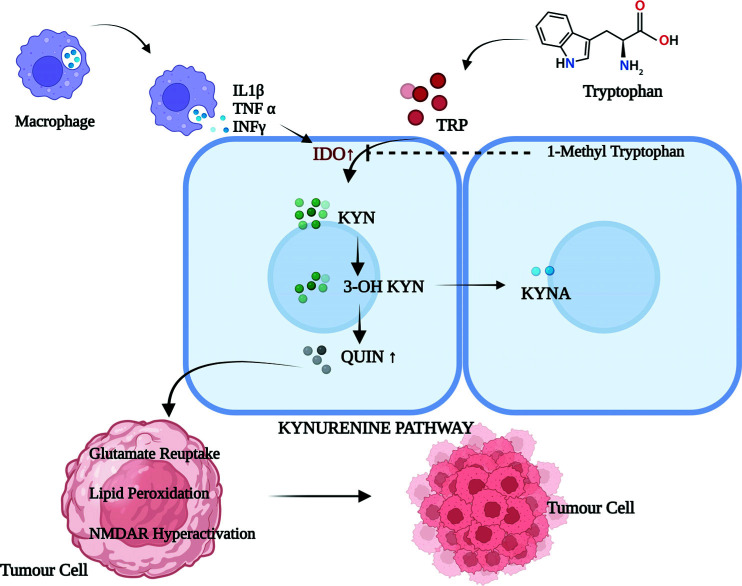
IDO as a checkpoint in the Kynurenine pathway. Legend: The IDO is the rate-limiting metabolite of the tryptophan metabolism, which is up-regulated by the proinflammatory cytokines IL 1β, TNF α, and INF γ, thus generating the toxic metabolites (Quinolinic acid) affecting the cancer metabolism. **Abbreviations:** (TRP: Tryptophan, KYNA: Kynurenic Acid, QUIN: Quinolinic Acid, IL 1β: Interlukine 1β, TNF α: Tumor Necrosis Factor α and INF γ: Interferon γ).

IDO is a rate-limiting enzyme in L-tryptophan catabolism, widely expressed in a variety of cells, and likely contributes to immune suppression and peripheral tolerance in mammals [80, 81]. IDO catalyses the first reaction in the kynurenine pathway [82] and produces a series of metabolic intermediates like quinolinic acid (QUIN), L-kynurenine, and picolinic acid (PIC), which can inhibit T and NK cells proliferation [83] Another intermediate substance, 3-hydrozykynurenine (3-HK), was found to induce the production of free radicals, which, in turn, directed the immunomodulation. However, a further increase in the catabolism of L-tryptophan by inflammatory IFN-γ induced intracellular IDO in monocytes and tumor cells, thus restraining the functions of NK and T-cells effectors [84]. Similarly, IDO overexpression in inflammatory bowel disease prevents the tissue-damaging activity of activated T-cells, unveiling its anti-inflammatory functions [85]. Furthermore, IDO expression in the placenta prevents allogenic fetus rejection during early pregnancy [86]. Likewise, IDO expression in tumor cells of the immunogenic murine model did not tolerate the rejection in the mice model, which was preimmunized with tumor antigen, due to the reduced T-cell infiltration to the tumor site [87] (Fig. **2**). However, IDO inhibition by an oral drug, 1-methyl-tryptophan or indoximod remarkably suppressed tumor succession. It is the only drug available against IDO and is currently under phase I/II clinical trials against complex extracranial solid tumors. Indoximod with the combination of conventional chemotherapies or radiation and with other checkpoint inhibitors, such as ipilimumab, pembrolizumab, or nivolumab, has shown grade 2 IRAE hypophysitis in 6% of pre-treated patients [88]. Likewise, other phase I/II trials have been testing indoximod against recurrent glioma (NCT02052648).

Though preclinical studies on IDO and indoximod against PBTs are limited, currently a phase I trial (NCT02502708) is focused on testing indoximod in combination with TMZ against primary malignant brain tumors of patients aged between 3-21 years. During this trial, the dosage of indoximod (12.8 to 22.4 mg/kg/dose) and one dose of TMZ (200 mg/m^2^) for a total of five days were given to measure the toxicity, responsiveness, pharmacokinetics, and progression-free survival benefits. Previously, IDO was examined in pediatric extracranial solid tumors, such as osteosarcoma, in which, patients’ samples scored ≥ 4 IDO expression measured by immunohistochemistry (IHC). Moreover, it demonstrated poor metastasis-free survival in 53% of patients compared to less-scored patients with 81% metastasis-free survival [89]. Furthermore, IDO expression was also noticed in 85% (17/20) of patients with pediatric Hodgkin lymphoma and Ewing sarcoma.

### 2.5 B7-H3

B7-H3 (CD276), an important immune checkpoint, belongs to the B7-CD28 family, having 28% homology with other molecules of the same family. Its coding gene is located on chromosome 15q23-q24 in humans and chromosome 9 in mice. It is a type-I transmembrane protein consisting of 534 amino acids with a molecular weight of 57 kDa. B7-H3 was first identified in humans [90], followed by mice [91]. However, its expression is universal across the species. The extracellular domain of B7-H3 is composed of a pair of Ig variable (V) and constant (C) domains without exon duplication in mice (2Ig B7-H3 isoform) and two identical pairs of IgV-IgC domains in humans (4Ig B7-H3 isoform; a predominant splicing variant) with exon duplication. It has no intracellular signalling motif in spite of its short tail [92-95]. Detectable levels of a soluble splicing variant of CD276 without a transmembrane domain were observed in human sera, which might be due to its cleavage from the surface by matrix metallopeptidase (MMP) or alternative splicing of introns [96]. B7-H3 mRNA levels were ubiquitously found in many tissues, like the liver, heart, prostate, spleen, and thymus. However, its protein expression was found to be limited at a steady state, suggesting the involvement of key post-transcriptional control mechanisms [97]. Furthermore, B7-H3 is likely to play a key role in the interaction between tumors and the immune system. It is a ligand for unknown T-cell receptors and binds to the T-cells with its FG-loop of the Ig variable domain.

Over the years, it has been speculated that B7-H3 may show functional dualism on T-cell mediated immune response by interacting with counter-receptors on T-cells [98-100]. B7-H3 has shown co-stimulatory functions like T-cell proliferation, cytokine production, and cytotoxicity by interacting with TREM (triggering receptor expressed on myeloid cell), like transcript 2 (TLT2) [101], which is a counter-receptor on CD8+ T-cells in mice but not in humans [101]. However, the majority of the outcomes have been correlated with its co-inhibitory function on T-cells. In this concern, B7-H3 expression on tumor cells targets NK-cells to suppress their mediated lysis of the tumor. Its wide expression on APCs inhibits T-cell function by diminishing the activity of NFAT, NF-kB, and AP-1 transcription factors [102]. Its expression on DCs stimulated by CD4+CD25+ Tregs, which impairs effector T-cell function [103], is evidence for its co-inhibitory role. Despite the controversial findings regarding its interaction with the other proteins and functions [104], it is still a challenge to identify its functional receptors in humans for better elucidation of CD276 downstream signaling in immune responses and tumor evasion [105, 106].

B7-H3 overexpression has been observed in a wide variety of human solid tumors, which correlates with negative or poor cancer prognosis. Moreover, inducible CD276 expression relatively correlates with the aggression of tumorigenesis and metastasis. Previous reports demonstrated the overexpression of B7-H3 in human malignancies, such as cutaneous melanoma [107], acute leukemia [108], breast cancer [109], prostate cancer [110], ovarian cancer [111], pancreatic cancer [112], colorectal cancer [113], GBM [114], and other cancers. B7-H3 is found on the membrane, in the cytoplasm, or within the nucleus of cancer cells, *e.g*. osteosarcoma [115] and colon cancer [116]. Interestingly, it was also found in tumor-associated vasculature in renal cell carcinoma [117]. Its overexpression increases the risk of disease spread during surgery, cancer recurrence, and ultimately death in prostate cancer patients [110]. Most recently, in breast cancer, B7-H3 has been successfully used as a biomarker for ultrasound molecular imaging [118]. Due to high differential expression between tumors and normal tissues, B7-H3 has emerged as a promising target for breast cancer therapy, including triple-negative breast cancer (TNBC) [119-122]. Breast cancer patients with B7-H3^high^/Foxp3^high^ tumors have shorter recurrence-free survival [123]. Abnormal glycosylation, mostly high rate of fucosylation in B7-H3 during oral cancer, may lead to DCs tolerance because membrane proteins like DC-specific ICAM-3-grabbing non-integrin (DC-SIGN) and Langerin of DCs have a better affinity with CD276 [124]. It has been observed that the relative expression of B7-H3 mRNA was higher in melanoma tissues [107] and blood specimens of gastric cancer patients compared to their healthy counterparts [125]. Additionally, an increased B7-H3 expression reduced TILs number and elevated lymph node metastasis, suggesting immune evasion and tumorigenesis in lung cancer [126]. Furthermore, tumor-associated macrophages (TAMs) derived IL-10 attenuated T-cell anti-tumor immunity in lung cancer mice model, which was correlated with increased expression of B7-H3 on the membrane [127]. Fibroblast-associated B7-H3 expression correlated with the shorter metastasis-free survival and the endothelial B7-H3 expression significantly correlated with poor outcome of colorectal cancer [116, 128]. However, its silencing by RNA interference (RNAi) technology was found to increase chemosensitivity of Ara-C and apparently reduce mRNAs of both MMP-2 and MMP-9 in acute myeloid leukemic cell line (U937), indicating B7-H3 involvement in transcriptional regulation of these two genes [129], which plays a central role in tumor migration, invasion, and metastasis [130].

The molecular mechanism behind the mode of action of B7-H3 remains unknown. However, the non-immunological functions, such as invasion, metastasis, and adhesion to fibronectin [131] of B7-H3 mediated by Jak2/Stat3/MMP-9 signaling [132], received great attention over its co-stimulatory or co-inhibitory effects on immune cells and the anti-tumor response in early studies. B7-H3 has no definite role in cell growth or proliferation [107]. In breast and colorectal cancers, overexpression of B7-H3 derails the apoptosis process by inducing Jak2/Stat3/Slug signalling, which further increases resistance to paclitaxel. Conversely, its knockdown in breast cancer reduces the phosphorylation of STAT3 Tyr705 residue *via* the inactivation of Jak2 [133, 134]. In hepatoma cells, B7-H3 induces epithelial-mesenchymal transition (EMT) *via* Jak2/Stat3/Slug signalling pathway [135]. In melanoma cells, B7-H3 alters the expression of metastasis markers like MMP-9, tissue inhibitor of metalloproteinase (TIMP)-1 and 2, STAT-3, and IL-8 [115, 131, 136, 137]. However, its knockdown downregulates pSTAT3 and cyclin D1, which are key molecules in the cell cycle [107]. The relationship between B7-H3 and metabolism was unveiled by identifying reduced glycolytic efficacy at low B7-H3 expression, which further increased the sensitivity of breast cancer cells to Akt/mTOR inhibitors [138]. Concurrently, another report demonstrated that B7-H3 promotes the Warburg effect (*i.e*., increased glucose uptake and lactate production) in breast cancer cells by inducing hypoxia-inducible factor (HIF)-1α and its downstream enzymes, *e.g*. lactate dehydrogenase (LDH)-A and pyruvate dehydrogenase kinase 1 (PDK1) of the glycolytic pathway, resulting in tumor growth [139].

Over the decade, vascular endothelial-specific B7-H3 expression on tumor cells becomes an attractive target for cancer immunotherapy. In this context, an unconventional mAb (Enoblituzumab/MGA271), which can operate antibody-dependent cell-mediated cytotoxicity (ADCC), was tested against B7-H3 in phase-I clinical trials on different cancers [122, 140, 141]. Likewise, a weekly dosage of MGA271 controlled the tumor growth and exhibited an Fc-mediated effect in renal and bladder carcinoma xenografts [122]. Its anti-tumor activity was also mediated by increasing T-cell repertoire clonality in peripheral blood.

8H9 is an antibody-drug conjugate that specifically binds to the FG loop of B7-H3 and has shown clinical success in patients with metastatic CNS neuroblastoma [142, 143]. Using small interfering (si)-RNA technology, Chen *et al.* demonstrated that downregulation of human B7-H3 reduced the cell adhesion to fibronectin, migration, and Matrigel invasion of melanoma and breast cancer cells. Other studies have also reported that B7-H3 was important in regulating the adhesion, migration, and invasion characteristics of pancreatic and prostate cancers, GBM, cutaneous melanoma, and osteosarcoma. These studies suggest that it may be a potential therapeutic target for the tumors that overexpress B7-H3. Small-hairpin (sh) RNA targeting B7-H3 in combination with the Ara-c drug was found to upregulate antineoplastic activity and cause 80% tumor reduction in histiocytic lymphoma compared to 40% with Ara-c alone [129]. Similarly, shRNA of B7-H3, together with paclitaxel, caused an 80% tumor reduction in breast cancer, thereby indicating shRNA-induced chemosensitivity and apoptosis in tumor cells [133]. Most recently, Shi *et al.* identified that the mAb Y4F11 was able to interact with the antigen B7-H3 on different immune cells from malignant pleural effusions of lung cancer patients grafted to BALB/c mice [144]. However, further therapeutic approaches like blocking mAbs, bispecific mAbs, chimeric-antigen receptor (CAR) T-cells, small molecule inhibitors, and synergistic options (chemo/radiation/ anti-CTLA4/anti-PD-1 together with anti-B7-H3) need to be evaluated in targeting B7-H3 for different cancers, including PBTs. MicroRNA (miR)-29a, 29b, and 29c inversely correlate with B7-H3 protein expression, and these miRNAs typically target 3’-UTR of B7-H3 for silencing. However, they were found to be downregulated in solid tumors, including neuroblastoma [145], melanoma (only miR-29c) [107], sarcomas, brain tumors, and tumor cell lines. However, the role of miRNAs in MB development is not yet studied and B7-H3 may have diversified effects based on the type of tumor origin.

Apart from these immune checkpoints, most recently, several other molecules have emerged due to their immunomodulatory properties in human cancers. The lymphocyte activation gene 3 (LAG-3) [146], fibrinogen-like protein 2 (FGL2) [147], colony stimulating factor-1 receptor (CSF-1R), T-cell immunoglobulin mucin domain (TIM)-3 [148], V-domain Ig suppressor of T cell activation (VISTA) [149], inducible co-stimulator (ICOS) [150], OX40 [151], and 4-1BB [152] have entered into clinical trials. The current combination immunotherapy in pediatric brain tumors is presented in Table **1**. However, further evaluation is required to register them in pediatric cancer therapy [153-164]. There is a requirement for concrete and robust research on tumor microenvironment and host-tumor interactions in pediatric cancers.

## 3 IMMUNOTHERAPY OF BRAIN CANCER AND CLINICAL TRIALS

**Fig. (3):**
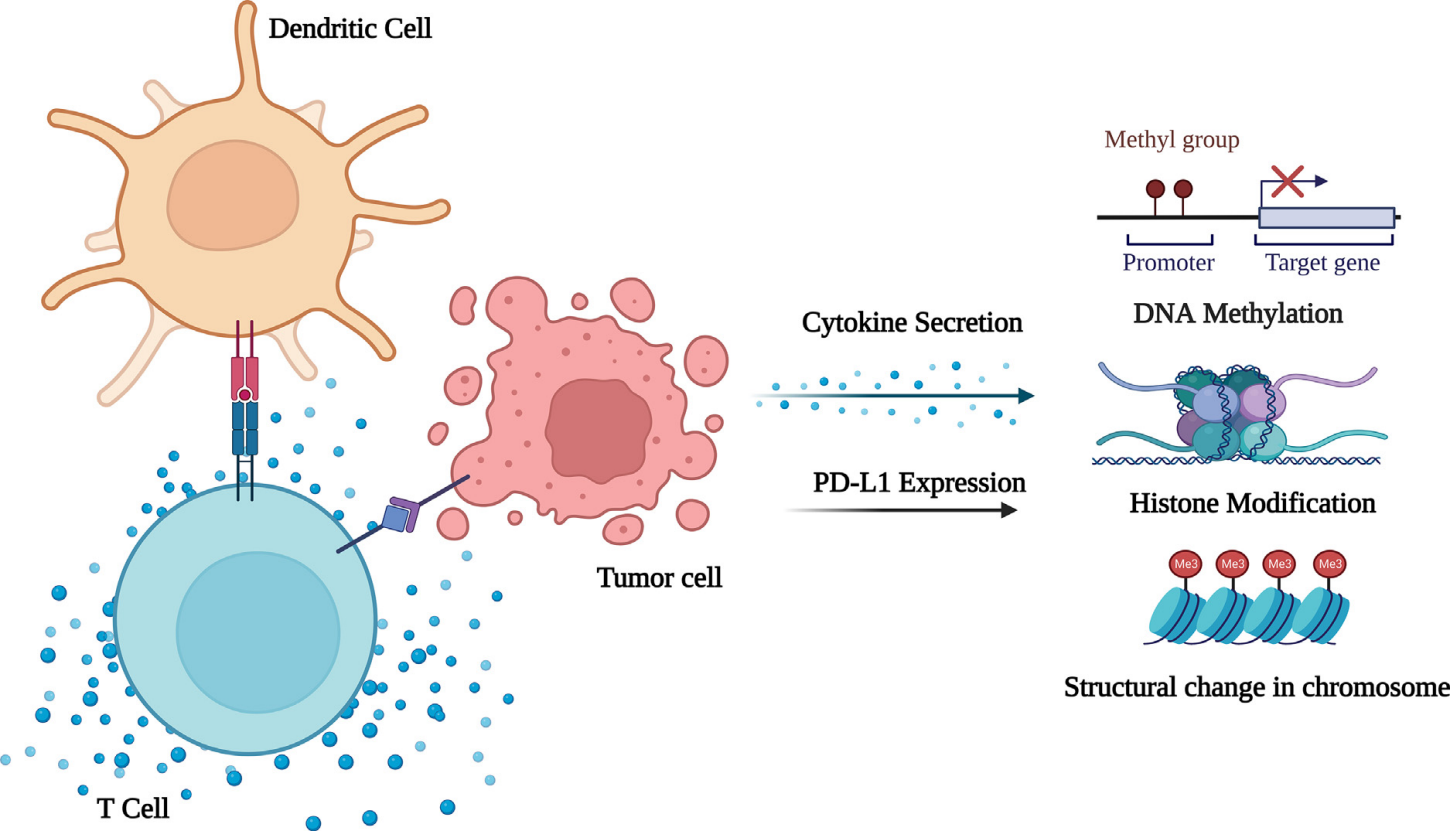
Cytokines influencing the epigenetics and cancer metabolism. Legend: After T-cells activation by the dendritic cells, they interact with the nearby cancerous cell and release cytokines. These cytokines then affect the epigenetics, such as DNA modification, histone modification, change in the structure of chromatin, *etc*.

It is known that tumors can escape from immune surveillance by exhibiting immune tolerance. However, this can be surpassed efficiently by different approaches: (I) Using mAbs [165] against immune checkpoints and tumor-specific antigens (TSAs), *e.g*. targeting epithelial growth factor receptor (EGFR)/EGFRvIII in GBM with ABT-414, an antibody-drug conjugate in phase I (NCT01800695) and II (NCT02573324) clinical trials and (II) Using tumor-specific or tumor-associated antigen vaccines [166], *e.g*. tumor-derived heat shock protein/protein complex (HSPPC)-96 vaccine, recently tested in phase II trials (NCT01814813) against surgically removable recurrent glioma and revealed its clinical benefit [167]. SL-701, a vaccine comprising multiple synthetic peptides, is in phase I/II trials, testing against recurrent GBM (NCT02078648). ICT-121, a CD133 pulsed-dendritic cell vaccine, is in phase I trials, testing against recurrent GBM (NCT02049489). A dendritic cell vaccine combined with imiquimod, which is a Toll-like receptor (TLR)-7/8 agonist, is in phase I trials, testing against adult and pediatric glioma (NCT01808820) as well as pediatric brain cancer (NCT01902771). Imiquimod combined with GBM-6, which can target brain tumor-initiating cells (BTICs), is in phase I trials, testing against pediatric DIPG (NCT01400672). ADU-623 targets EGFRvIII and NY-ESO-1 antigens and is in phase I trials, testing against recurrent grade III/IV astrocytomas (NCT01967758). Further pilot studies have been testing glioma antigen peptides in combination with imiquimod and Poly-ICLC (Hiltonol^®^), which is a TLR-3 agonist against recurrent ependymomas (NCT01795313) and pediatric glioma (NCT01130077), respectively. Apart from these, glioma-associated antigens like EphA2, IL-13Rα2, and Survivin can be potential vaccine candidates against pediatric brainstem and non-brainstem glioma [166]. (III) Targeting cytokines and inflammatory pathways [168], *e.g*. targeting IL-2, IL-4, IFN-γ, TNF-α, and others that are reluctant to treat brain tumors [169] (Fig. **3**). (IV) Adoptive cell therapy, in which genetically altered immune cells are reinfused into the patients with the target of improving anti-tumor immunity, such as testing anti-EGFRvIII CAR T-cells against malignant glioma (NCT01454596) and GBM (NCT02209376) is in phase I/II clinical trials.

## 4 EPIGENETIC LANDSCAPE OF HISTONE H3 AND POSSIBLE ROLE IN BRAIN TUMORS

**Fig. (4):**
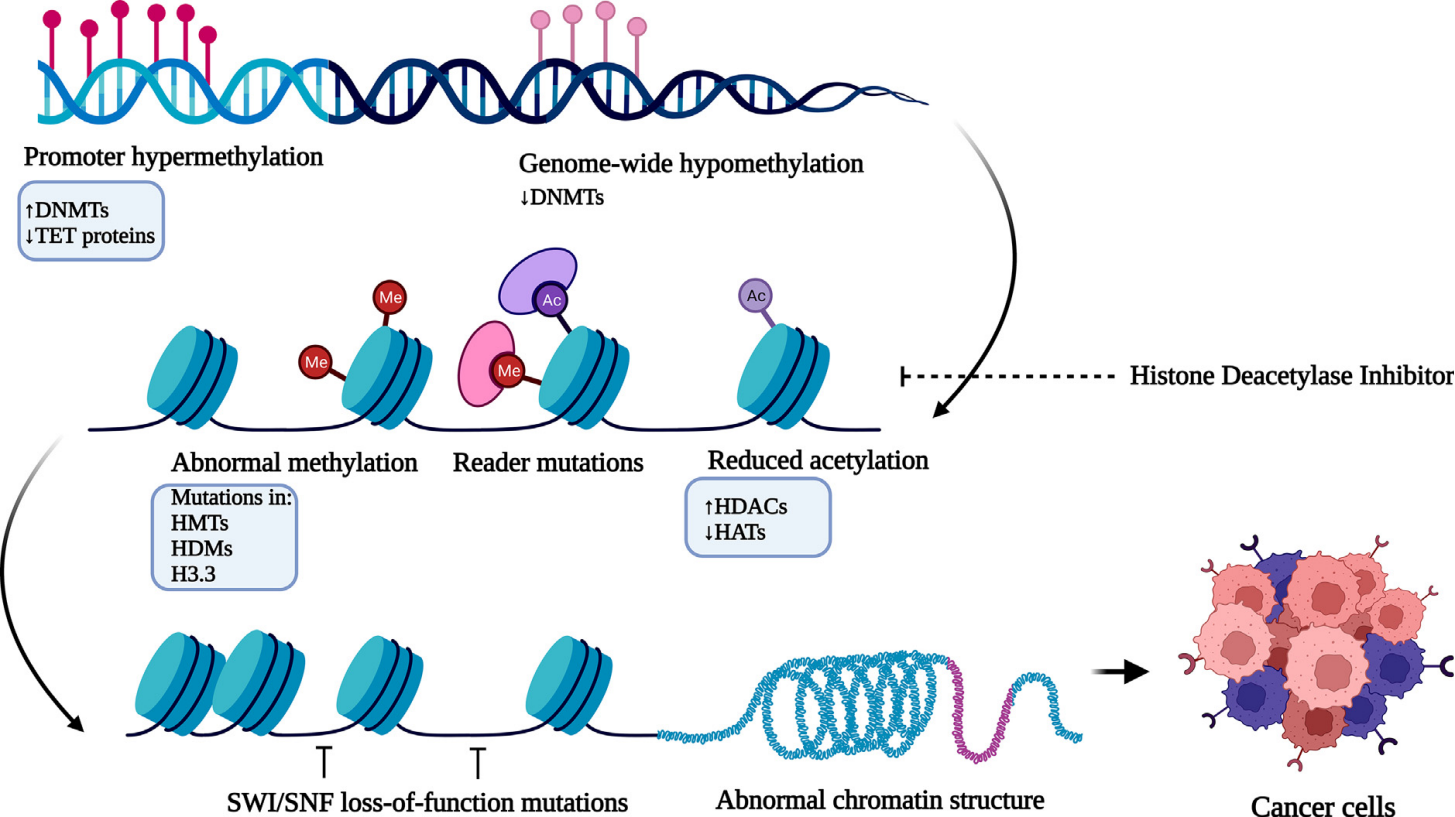
The epigenetics of the histone protein modification. Legend: Histone protein modification caused by the hypermethylation, mutation, and reduced acetylation affecting the SWI/SFN complex is responsible for alteration in the structure of the chromatin, thereby affecting the cancer metabolism **Abbreviations:** (SWI/SEN: switch/sucrose non-fermentable complex, HDMs: Histone demethylase, DNMT: DNA Methyl Transferase, HMTs: Histone demethyltransferase, HAT: Histone acetyltransferase, HDAC: Histone deacetylase, TET: Ten Eleven Translocation).

The nuclear chromatin packing is finely coordinated by histone proteins *via* certain PTMs, including acetylation, methylation, phosphorylation, sumoylation, poly (ADP)-ribosylation, and ubiquitination. With these modifications, histones become highly dynamic in regulating replication, transcription, and DNA repair. For instance, histone H3 trimethylation of lysine 4 (H3K4me3) and acetylation of lysine 9 (H3K9ac) are associated with biologically active genes. Conversely, histone H3 dimethylation of lysine 9 (H3K9me2), H3K9me3, H3K27me2, and H3K27me3 is linked with inactive genes, denoting the overlapping pattern of histone methylation. In embryonic stem cells, the bivalent histone marks are speculated to maintain the genes that remain transcriptionally active. They are mediated by inducing epigenetic silencing *via* DNA hypermethylation in a few heritably regulatory genes, which may result in malignant transformation or tumor succession [170]. In fact, these modification patterns are regulated in a manner by the enzymes, *e.g*. histone acetyltransferases (HATs) and histone methyltransferases (HMTs), which typically transfer acetyl and methyl groups from the sources, such as acetyl-CoA and S-adenosyl methionine (SAM), respectively. Conversely, histone deacetylases (HDACs) and HDMs excise acetyl and methyl groups, respectively [171, 172]. The dysregulation of these enzymes in cancer becomes a potential target for epigenetic therapy. For example, altered expression of class II and IV HDACs seems to be potential targets for GBM treatment [173] (Fig. **4**). Furthermore, high-throughput large-scale gene sequencing of GBM patients revealed other mutations in several protein-coding genes, which are typically involved in epigenetic regulation, *e.g*. HDAC 2 and 9, HDMs (JMJD 1A and 1B), and HMTs (SET 7 and SETD7, MLL and MLL4, and MBD1). However, their role in gliomagenesis is not yet established [174]. Mutations in PTMs of histones, for example, significant down-regulation of the MLL-p27 (Kip1) pathway in pituitary adenomas and up-regulation of BMI1, which is a component of polycomb repressive complex 1 (PRC1) in MB, are known to have a high rate of mortality or poor survival [175, 176].

**Fig. (5):**
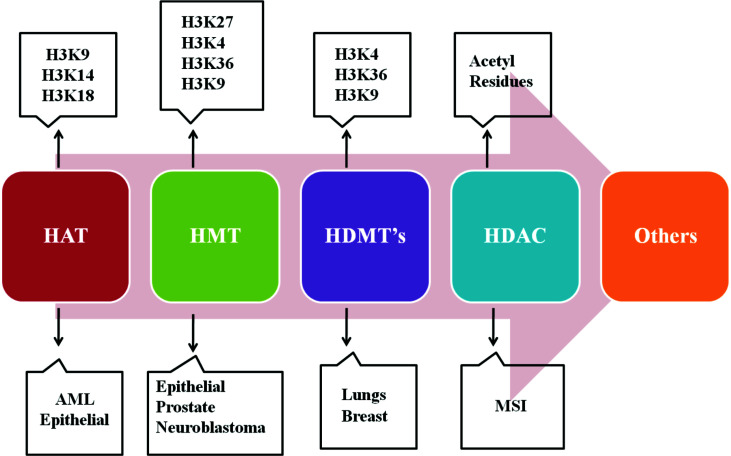
Epigenetic landscape of histone H3 in the pediatric brain tumor. Legend: The epigenetic modification in the various genes reacting with the H3 histone protein, thus affecting the tumor progression. Different classes of histone protein H3 affecting different tumors. AML: Acute myeloid leukaemia, MSI: Microsatellite instability.

Until mid of this decade, studies on histone mutations in human cancers are very limited. Later, they were studied exclusively in PBTs but not in the GBM of elderly patients [177]. In non-brainstem pediatric GBM (non-BSPG) and DIPG, the genes encoding histone variants, such as H3.3 (H3F3A) and H3.1 (HIST1H3B and 3C) [178], have experienced remarkable recurrent somatic heterozygous mutations. In which, mostly the amino acid substitutions occurred; for example, K27M (lysine to methionine at position 27) in both H3.1 and H3.3 variants and G34V/G34R (glycine to valine or arginine at position 34) in H3.3 were identified in 78% of DIPGs and 36% of non-BSPGs (Fig. **5**). Interestingly, H3.3K27M mutations were largely detected in the thalamus, brainstem, cerebellum, and spinal cord, H3.3G34R/V mutations were exclusively distributed in cerebral hemispheres (cortex), and H3.1K27M mutations were confined to the pons; these are the exploring divergent transcriptional profiles and microenvironments engaged by different H3 mutations [179-182]. Despite the structural similarity between these two histone variants, they differ in five amino acids and their time of action. Since H3.1 plays a crucial role in the S-phase of cell cycle and is defined as replication-dependent, whereas, H3.3 is committed to being involved throughout the cell cycle and substitutes old H3 variants at selective loci throughout the genome [183] (Table **2**).

The predominance of selective mutations in histone variants in a large group of patients endows the importance of those amino acid residues in transcriptional regulation. Here, K27 of histone H3 has a key role in normal brain development, which frequently undergoes trimethylation (H3K27me3) using methyltransferase activity of enhancer zeste human homolog 2 (EZH2) of PRC2, which directs to inhibit the transcription of various genes (*e.g*. X-chromosome) involved in lineage assertion, differentiation, and devising of anterior-posterior ends [194, 195]. Although the H3K27M mutation in these PBTs competitively inhibits EZH2 methyltransferase activity, the PRC-mediated transcriptional silencing of a variety of genes is aborted [196]. Obliquely, EZH2 may be capable of regulating DNA methylation by serving as a platform to recruit DNMTs [197]. Furthermore, EZH2 overexpression has been reported in various brain tumors, including astrocytoma, oligodendroglioma, and also in GBM [198]. Another plausible similar mutation was identified, H3K27I, in which, the transversion occurs from ‘AAA’ to ‘ATA’ codon, which codes for inosine instead of lysine and exhibits slightly inhibitory activities on PRC2 and H3K27me3. However, further investigations are still required into PBTs [199].

Conversely, H3G34R/V mutation causes functional aberrations, which is not yet well understood, but it could affect the methylation pattern of H3K36 by negatively regulating the SETD2 methyltransferase domain of EZH2, which is specific to H3K36 [196]. Due to the proximity between G34 and K36 of histone H3, the H3G34R/V mutation alters the transcriptional process of certain target genes. Both these mutations of H3 (H3K27M and H3G34R/V) have different gene expression patterns themselves and when compared to the normal brain tissue. During these mutations, transcriptional activation of certain oncogenes or oncomiRs and repression of TSGs takes place, which, in turn, induces tumorigenesis. Likewise, other variants of similar mutations are also reported in rare childhood bone tumors, such as H3F3A G34W/L (glycine to tryptophan/leucine at position 34) in giant cell tumors and H3F3B K36M in chondroblastoma [200]. It has been observed that both histone H3.3 and H3.1 mutations substantially reduce the DNA methylation right through the epigenome. The K27M mutation reduces DNA methylation globally and the G34R/V mutation predominantly reduces DNA methylation in subtelomeric regions [201].

Besides these histone H3 mutations, there are several protein-coding genes, *e.g*. ATRX and DAXX, which are involved in chromatin remodelling. These protein-coding genes frequently undergo mutations, which results in their inactivation [202]. Furthermore, they interfere with H3.3 incorporation at the heterochromatic loci of pericentric and telomeric regions of chromosomes in a replication-independent manner, ultimately resulting in the loss of structural integrity of chromosomes. Parenthetically, during ATRX mutation, the length of the telomere sequence is successfully maintained, though there is no participation of telomerase. Hence, it is called alternative lengthening of telomeres (ALT), which facilitates genome stability and growth in tumor cells [202, 203]. Similar mutations were also noticed in pediatric and adult GBMs, neuroblastomas, and pancreatic neuroendocrine tumors.

## 5 HDIs IN THE TREATMENT OF BRAIN CANCER

Indeed, acetylation and deacetylation modifications of histones regulate chromatin remodelling. However, in cancer progression, abnormal deacetylation causes transcriptional inactivation of TSGs due to chromatin condensation, resulting in epigenetic silencing of TSGs. However, HDIs can able to nullify this process in different types of tumors by promoting the re-expression of various genes, which are involved in differentiation and growth arrest or apoptosis [204]. Concurrently, several HDIs, including vorinostat or SAHA (suberoylanilide hydroxamic acid), depsipeptide, and valproic acid, have entered into clinical trials. The SAHA was shown to arrest the cell cycle at the G2/M transition phase, increase pro-apoptotic and anti-proliferative genes expression (*e.g*. DR5, TNF-α, p21WAF1, p27KIP1), and decrease anti-apoptotic and pro-growth genes expression (*e.g*. CDK2, CDK4, cyclin D1, cyclin D2) in GBM cells [205]. Children’s Oncology Group conducted phase I clinical trials and demonstrated that vorinostat was safely tolerable when used alone or in combination with 13-cis retinoic acid (RA) [206]. SAHA combined with RA had an additive effect on the induction of BMP-2 transcription, p38MAPK independent apoptosis in MB, and cisplatin sensitivity because both drugs have the ability to cross the blood-brain barrier (BBB) [207]. Other preclinical studies on vorinostat have demonstrated its anti-tumor activity in MB [207-209] and HGGs [205]. However, further clinical trials on vorinostat with the combination of chemo/radiotherapy are in progress against pediatric gliomas, DIPG, and MB.

Valproic acid was initially recommended as an anti-epileptic drug and further enrolled by HDI activity. It was found to have a potential role in adjuvant radio- and chemo-therapy against HGGs and was safely tolerable [210-212]. Simultaneously, it improved histone acetylation in PMBCs of 50% of the patients at the dosage of 75-100 μg/mL. Though it has shown pulmonary embolism as an adverse effect, it thrives for better survival in anaplastic astrocytoma compared to GBM. The utility of depsipeptide in pediatric tumors remains to be unknown because it does not exert an anti-tumor response but improves histone acetylation, as studied in an *in-vivo* cancer model [213]. Likewise, romidepsin was also inefficient against recurrent GBM at a single standard dose and schedule [214]. Other HDIs, including entinostat, panobinostat, and phenyl-butyrate, are currently in a clinical evaluation state against HGGs or refractory PBTs and neuroblastoma. In addition, HDIs can induce sensitivity in drug-resistant cells, suggesting that drug resistance can be reversed by HDIs. At the end of 2015, a phase I clinical trial was started by PBT Consortium (PBTC047) to test panobinostat (HDI) against children with DIPG. On the other hand, drugs targeting histone methylation are in preclinical standard, which are likely to be interfering with PRC2 components. The SL11144 drug targets lysine (K)-specific demethylase 1A (KDM1A) and DZNep, which inhibits S-adenosyl homocysteine hydrolase [20]. Likewise, a phase II study is currently investigating 131-iodine (I^131^) conjugated mAb (Cotara) against DNA-histone H1 complexes *via* an advanced delivery system in recurrent GBM [215]. Moreover, it has been observed that HDI inhibitors act as a therapeutic treatment for pediatric brain cancer (Table **3**).

## 6 CURRENT STATUS OF SELECTED DRUGS FOR PEDIATRIC BRAIN CANCER

According to clinical research, the majority of pediatric brain tumors still lack a viable treatment. The gap in research is due to the limited understanding of the biology of brain tumors. The translation of these drugs from the preclinical stage to clinical testing has been challenging due to similar reasons [226]. The network for research on brain tumors is being unified in nascent stages. Over a long period of time, the international pediatric brain tumor community has shown remarkable collaborative activity. Currently, the community is working to identify the genomic subtypes of pediatric brain tumors and to create multidisciplinary teams to conduct preclinical studies that will better guide the design of clinical trials [227]. Such trials may offer potential possibilities to change the current research direction and incorporate the examination of new ideas (Table **4**) [228]. Despite these obstacles, the community's immediate goal is to improve the way clinical trials are now conducted. International collaborative clinical trials are essential in the setting of limited, gnomically defined patient subgroups. It is, thus, necessary to conduct a thorough analysis of the present administrative, legal, and financial barriers that prevent the start of such trials [229].

## CONCLUSION

These checkpoints provide distinct insights into auto immunity and robust antitumor response. The significant evidence points to immunological check point inhibitors as a promising therapeutic option for pediatric cancers based on considerable responses reported in a number of trials examining their effects on pediatric malignancies. Similarly, checkpoint inhibitors, alone or in conjunction with drugs that block the histones' epigenetic landscape, might be more effective in treating pediatric refractive solid brain tumors. Immune checkpoint inhibition is a possible treatment for pediatric cancers based on strong responses shown in a variety of pediatric malignancies and early data showing that many pediatric solid tumors express checkpoint molecules. Studies on adult checkpoint inhibitors revealed some notable, potentially fatal autoimmune side effects, with combined PD-1 and CTLA-4 blockers exhibiting greater toxicity. Even though histone deacetylase inhibitors have shown therapeutic efficacy in mitigating pediatric brain tumors, the hypothesis of using histone deacetylase inhibitors needs further exploration using preclinical and clinical studies. On a similar aspect, the use of immunotherapy and other histone deacetylase inhibitors providing therapeutic efficacy in mitigating pediatric brain tumors should be further explored in this field. In this concern, we speculate that more research is necessary for evaluating the efficacy and safety of combined targeting B7-H3 and H3K27me3 in recurrent PBTs for future prospects.

## Figures and Tables

**Table 1:** Drugs and their mechanism as immune checkpoint inhibitors.

S. No.	Drug	Class	Rationale of Uses	References
1.	Nivolumab (Opdivo)	PD-1 inhibitors	Gastro-esophageal adenocarcinoma	[153]
2.	Cemiplimab (Libtayo)	PD-1 inhibitors	Advance cutaneous squamous-cell carcinoma	[154]
3.	Atezolizumab (Tecentriq)	PD-L1 inhibitors	Small- cell lung cancer	[155]
4.	Avelumab (Bavencio)	PD-L1 inhibitors	Advanced urothelial carcinoma	[156]
5.	Durvalumab (Imfinzi)	PD-L1 inhibitors	Biliary tract cancer	[157]
6.	Ipilimumab (Yervoy)	Cytotoxic T-lymphocyte antigen-4 (CTLA-4)	Advanced melanoma	[158]
7.	Tremelimumab (Imjuno)	Cytotoxic T-lymphocyte antigen-4 (CTLA-4)	Hepatocellular carcinoma	[159]
8.	Relatlimab	Lymphocyte Activation Gene-3 (LAG3; CD223)	Chronic lymphocytic leukemia (CLL)	[160]
9.	Opdualag	Lymphocyte Activation Gene-3 (LAG3; CD223)	Melanoma	[161]
10.	Indoximod	Indoleamine 2,3-dioxygenase (IDO-1) inhibitors	Advanced or metastatic melanoma	[162]
11.	Epacadostat	Indoleamine 2,3-dioxygenase (IDO-1) inhibitors	Renal cell carcinoma (RCC)	[163]
12.	Navoximod	Indoleamine 2,3-dioxygenase (IDO-1) inhibitors	Recurrent advanced solid tumors	[164]

**Table 2:** Epigenetic landscape of histone 3 in the pediatric brain tumor.

**Enzyme Category**	**Gene Name**	**Histone Substrates**	**Defect**	**Tumor Type**	**References**
HAT	CBP (KAT3A)	H3K18, H3K14	Translocation	AML	[184]
	pCAF (KAT2B)	H3K9, H3K14, H3K18	Mutation	Epithelial Cancer	[185]
HMT	EZH2 (KMT6)	H3K27	Amplification Mutation	Prostate Lymphoma	[186]
	MLL1 (KMT2A)	H3K4	Translocation	Leukemia	[187]
	NSD1 (KMT3B)	H3K36	CpG hypermethylation	Neuroblastoma, glioma	[188]
	RIZ1 (KMT8)	H3K9	CpG hypermethylation	Breast	[189]
Histone demethylase (HDMTs)	GASC1 (KDM4C)	H3K9, H3K36	Amplification	ESCS; lung; breast	[190, 191]
	LSD1 ((KDM1)	H3K4, H3K9	Amplification	Prostate; bladder; lung; CRC	[192]
Histone deacetylases (HDACs)	HDAC2	Many acetyl residues (except H4K16)	Mutation	MSI	[193]

**Table 3:** Current status of selected drugs, HDI inhibitors, for pediatric brain cancer.

**S. No.**	**Drug**	**Developer**	**Country of Origin**	**Rationale of Use**	**Current ** **Status**	**References**
1.	CUDC-907	Dana-Farber Cancer Institute/Boston Children's Hospital, Boston	United States	Solid tumors, CNS tumors, and Lymphomas	Phase 1	[216]
2.	Marizomib+ Panobinostat	Boston Children's Hospital Boston, Massachusetts, United States Dana-Farber Cancer Institute Boston, Massachusetts, United States	United States	Diffuse Intrinsic Pontine Glioma Pediatric Brainstem Glioma Acute myeloid leukemia	Phase 1	[217]
3.	Panobinostat	Department of Neurology, Stanford University, Palo Alto, CA 94305, USA	United States	Diffuse Intrinsic Pontine Glioma	Phase 2	[218]
4.	Entinostat	Children's Hospital of Alabama, United states	United States	Brain Stem Neoplasm Pineal Region Neoplasm	Phase 1	[219]
5.	Valproic acid	Texas Children's Cancer Center, Texas Children's Hospital, Baylor College of Medicine	United States	Medulloblastoma and supratentorial primitive neuroectodermal tumor	Phase1	[220]
6.	Sodium butyrate	Brain Tumor Research Laboratory, Department of Neurosurgery, University of Illinois at Chicago	United States	Gliomas	Pre-clinical	[221]
7.	Trichostatin A (TCA)	Genome Stability Laboratory, Department of Physiology, Yong Loo Lin School of Medicine, National University of Singapore, Singapore 117597, Republic of Singapore	Republic of Singapore	Medulloblastoma	Pre-clinical	[222]
8.	Suberoyl anilide hydroxamic acid (vorinostat)	Texas Children’s Cancer Center, Baylor College of Medicine, Houston, Texas, USA;	USA	Diffuse intrinsic pontine glioma	Phase 2	[223]
9.	Belinostat	Huntsman Cancer Institute	USA	Brain tumor	Phase 1	[224]
10.	Romidepsin	Children's Cancer Centre Research Laboratory, Murdoch Children's Research Institute, Royal Children's Hospital, Parkville, Australia	Australia	Atypical teratoid rhabdoid tumor	Pre-clinical	[225]

**Table 4:** Current status of selected drugs for pediatric brain cancer.

**S. No.**	**Drug**	**Developer**	**Country of Origin**	**Rationale of Use**	**Current Status**	**References**
1.	Dabrafenib	Pediatric Oncology Unit, UCL Great Ormond Street Institute of Child Health, London, United Kingdom	London, United Kingdom	In low-grade gliomas	Phase I/II trial	[230]
2.	Vemurafenib	Department of Neurological Surgery, University of California, San Francisco, San Francisco, CA, USA	San Francisco, CA, USA	Metastatic melanoma	Phase I	[231]
3.	Indoximod	Augusta University, Georgia Cancer Center	Georgia, United States	Glioblastoma, Medulloblastoma, Ependymoma, Diffuse Intrinsic Pontine Glioma	Phase 2	[232]
4.	MGMT(P140K)gene therapy	Children’s Cancer Research Unit, Kid’s Research Institute, The Children’s Hospital at Westmead, NSW, Australia	Australia	Pediatric brain tumor	Phase I	[233]
5.	Procarbazine, lomustine and vincristine	Department of Internal Medicine, University of Kentucky College of Medicine, Lexington, Kentucky, USA	Kentucky, USA	High-grade glioma	Phase I	[234]
6.	Nelarabine	Carilion Clinic, Pediatrics, Roanoke	USA	T-cell Malignancy	Phase 2	[235]
7.	Blinatumomab	Johns Hopkins University, Department of Oncology, Baltimore, USA	USA	Pediatric B-Precursor Acute Lymphoblastic Leukemia (B-ALL)	Phase 3	[236]
8.	Quinapril	St. Jude Children's Research Hospital, Pathology, Memphis, USA	USA	Acute Lymphoblastic Leukemia	Phase 1	[237]
9.	Mafosfamide	Departments of Pediatrics, Neurosurgery, Neuropathology and Radiotherapy, University of Vienna, Vienna, Austria	Austria	Embryonal tumors	Phase 1	[238]
10.	Volasertib	Institute Curie and University Paris Descartes, Pediatric, Adolescent and Young Adults Oncology Department, Paris, France	Paris, France	Acute Leukemia and Advanced Solid Tumors	Phase 1	[239]

## LIST OF ABBREVIATIONS

PBTs: Pediatric Brain Tumors
CTLA-4: Cytotoxic T-lymphocyte-associated Antigen
PD-1: Programmed Death
H3K27ac: Histone 3 Lysine 27 Acetylation
BT: Brain Tumors
MB: Medulloblastoma
ATRT: Atypical Teratoid Rhabdoid Tumor
DIPG: Diffuse Intrinsic Pontine Glioma
PNET: Primitive Neuroectodermal Tumor
MHC: Major Histocompatibility Complex
TCR: T-cell Receptor
MDSCs: Myeloid-derived Suppressor Cells
DNMTs: DNA Methyltransferases
TSG: Tumor Suppressor Gene
MBD: Methyl-CpG Binding Domain
PTMs: Posttranslational Modifications
FDA: Food and Drug Administration
HDIs: Histone Deacetylase Inhibitors
GBM: Glioblastoma
MGMT: Methylated O^6^-methylguanine DNA-methyl-transferase
TMZ: Temozolomide
GST: Gastrointestinal Tract
IDO: Indoleamine 2,3-dioxygenase
TREM: Triggering Receptor Expressed on Myeloid Cell
TAMs: Tumor-associated Macrophages
TNBC: Triple-negative Breast Cancer
TIMP: Tissue Inhibitor of Metalloproteinase
LDH: Lactate Dehydrogenase
HIF: Hypoxia-inducible Factor
PDK1: Pyruvate Dehydrogenase Kinase 1
ADCC: Antibody-dependent Cell-mediated Cytotoxicity
CAR: Chimeric-antigen Receptor
LAG-3: Lymphocyte Activation Gene 3
FGL-2: Fibrinogen-like Protein 2
VISTA: V-domain Ig Suppressor of T-cell Activation
EGFR: Epithelial Growth Factor Receptor
HATs: Histone Acetyltransferases
HDACs: Histone Deacetylases
BTICs: Brain Tumor-initiating Cells
PRC1: Polycomb Repressive Complex 1
EZH2: Enhancer Zeste Human Homolog 2
ALT: Alternative Lengthening of Telomeres
SAHA: Suberoylanilide Hydroxamic Acid
BBB: Blood-brain Barrier
